# Using Implementation Science to Support a Research and Public Policy Sector Older Adult Social Housing Partnership

**DOI:** 10.1093/geroni/igaa057.365

**Published:** 2020-12-16

**Authors:** Sander Hitzig, Christine Sheppard, Andrea Austen

**Affiliations:** 1 Sunnybrook Hospital, North York, Ontario, Canada; 2 Sunnybrook Research Institute, Toronto, Ontario, Canada; 3 City of Toronto, Toronto, Ontario, Canada

## Abstract

One quarter of the residents in the City of Toronto is comprised of older adults, and this number is expected to continue to grow dramatically over the next few decades. The development of evidence-based interventions to meet the health and social care needs of Toronto’s aging population can be hampered by failing to account for broader implementation considerations that can adversely affect successful uptake. The present initiative provides a case-example of a research and public policy sector partnership that used an implementation approach to co-design an older adult social housing model for low-income older adult groups. Implementation science is the study of the uptake of research evidence into practice. Our team used the Consolidated Framework for Implementation Research (CFIR) to support the planning, implementation and evaluation process of a new social housing model for older adults by: 1) identifying all relevant stakeholders; 2) generating evidence via qualitative interviews/focus groups, a scoping review, secondary data analysis, and an environmental scan; 3) facilitating large scale stakeholder consultation events with older adults, front-line practitioners and other community agencies; 4) supporting the development of an evaluation framework; and 5) providing opportunities for knowledge exchange and transfer across each phase of the initiative. An implementation science approach has augmented the ability of the City of Toronto to optimize the co-creation of housing strategies aimed at improving the overall wellness of vulnerable older adults living in social housing. Further, a number of valuable lessons were learned on how to foster successful research and public policy relationships.

